# Validation and Clinical Evaluation of a Novel Method To Measure Miltefosine in Leishmaniasis Patients Using Dried Blood Spot Sample Collection

**DOI:** 10.1128/AAC.02976-15

**Published:** 2016-03-25

**Authors:** A. E. Kip, H. Rosing, M. J. X. Hillebrand, S. Blesson, B. Mengesha, E. Diro, A. Hailu, J. H. M. Schellens, J. H. Beijnen, T. P. C. Dorlo

**Affiliations:** a Department of Pharmacy & Pharmacology, Antoni van Leeuwenhoek Hospital/Slotervaart Hospital, Amsterdam, the Netherlands; b Division of Pharmacoepidemiology & Clinical Pharmacology, Utrecht Institute for Pharmaceutical Sciences, Faculty of Science, Utrecht University, Utrecht, the Netherlands; c Drugs for Neglected Diseases Initiative, Geneva, Switzerland; d Leishmaniasis Research and Treatment Center, University of Gondar, Gondar, Ethiopia; e Department of Internal Medicine, University of Gondar, Gondar, Ethiopia; f Department of Microbiology, Immunology, and Parasitology, School of Medicine, Addis Ababa University, Addis Ababa, Ethiopia; g Department of Clinical Pharmacology, Antoni van Leeuwenhoek Hospital/Netherlands Cancer Institute, Amsterdam, the Netherlands; h Pharmacometrics Research Group, Department of Pharmaceutical Biosciences, Uppsala University, Uppsala, Sweden

## Abstract

To facilitate future pharmacokinetic studies of combination treatments against leishmaniasis in remote regions in which the disease is endemic, a simple cheap sampling method is required for miltefosine quantification. The aims of this study were to validate a liquid chromatography-tandem mass spectrometry method to quantify miltefosine in dried blood spot (DBS) samples and to validate its use with Ethiopian patients with visceral leishmaniasis (VL). Since hematocrit (Ht) levels are typically severely decreased in VL patients, returning to normal during treatment, the method was evaluated over a range of clinically relevant Ht values. Miltefosine was extracted from DBS samples using a simple method of pretreatment with methanol, resulting in >97% recovery. The method was validated over a calibration range of 10 to 2,000 ng/ml, and accuracy and precision were within ±11.2% and ≤7.0% (≤19.1% at the lower limit of quantification), respectively. The method was accurate and precise for blood spot volumes between 10 and 30 μl and for Ht levels of 20 to 35%, although a linear effect of Ht levels on miltefosine quantification was observed in the bioanalytical validation. DBS samples were stable for at least 162 days at 37°C. Clinical validation of the method using paired DBS and plasma samples from 16 VL patients showed a median observed DBS/plasma miltefosine concentration ratio of 0.99, with good correlation (Pearson's *r* = 0.946). Correcting for patient-specific Ht levels did not further improve the concordance between the sampling methods. This successfully validated method to quantify miltefosine in DBS samples was demonstrated to be a valid and practical alternative to venous blood sampling that can be applied in future miltefosine pharmacokinetic studies with leishmaniasis patients, without Ht correction.

## INTRODUCTION

Miltefosine is currently the only oral drug for both cutaneous leishmaniasis (CL) and visceral leishmaniasis (VL), and new studies to evaluate the use of miltefosine-based combination therapies in VL patients and in HIV-coinfected VL patients are under way (1). Recently, it was discovered that miltefosine treatment failure was associated with lower levels of drug exposure; the time that miltefosine plasma concentrations were >10 times the 50% effective concentration (17.9 μg/ml) was correlated with final treatment failure or success (2). This finding emphasizes the need for adequate pharmacokinetic (PK) monitoring in such clinical trials.

Both CL and VL are poverty-related diseases that mainly affect populations in resource-poor and remote regions of Africa, Asia, and South America. Classically, human blood plasma is collected by venous sampling for the measurement of drug concentrations, e.g., employing liquid chromatography-tandem mass spectrometry (LC-MS/MS). A bioanalytical method to quantify miltefosine levels in plasma was validated and reported previously (3). However, technologies such as LC-MS/MS are not available in the regions in which VL is endemic; therefore, samples need to be transported to appropriate facilities for analysis. The required cold storage (3) and transport of these plasma samples are logistically highly challenging, as well as expensive. In addition, plasma sampling by venipuncture is an invasive and risky sampling method, particularly for severely weakened and anemic HIV-coinfected VL patients. A large proportion of VL patients in East Africa are pediatric (4), which limits both the total volume and the number of plasma PK samples that can be obtained through venous blood sampling. Dried blood spot (DBS) sampling is an attractive alternative to plasma sampling in such settings because it is minimally invasive and requires only a small volume of blood (5–9), which is particularly advantageous in pediatric studies (10, 11). In addition, storage and shipment at room temperature are possible and therefore would be simple and low cost, which is preferred in remote areas without proper laboratory facilities.

Major hurdles in the application of DBS sample collection are the effects of hematocrit (Ht) levels and blood spot volumes on miltefosine quantification (12–14). Ethiopian VL patients had decreased median Ht levels of 25% (range, 23 to 30%) at the initiation of treatment (15), which slowly moved toward Ht levels of 33% (range, 27 to 37%) after 30 days of treatment with sodium antimony gluconate (15). HIV-coinfected VL patients showed similar Ht values during active VL infections (mean hemoglobin concentration of 9 g/dl, corresponding to a Ht value of approximately 27% [16]). Since miltefosine has a long terminal half-life (30.9 days) (17) and accumulates during treatment, pharmacokinetic sampling is typically performed at various time points during treatment and up to several months after the end of treatment. Ht values show high within-subject variability within this period, which may influence the outcomes of drug measurements with DBS sample collection.

Additionally, blood spot volumes can vary widely between patients, due to variations in blood flow and the penetration of the lancet in the finger. The viscosity of the blood increases with increased Ht levels (18); therefore, the blood flow and possibly blood spot volumes can be expected to be larger for patients with lower Ht levels.

Here we describe the development and validation of a rapid LC-MS/MS method to quantify miltefosine levels in DBS samples in a range from 10 to 2,000 ng/ml, according to the current Food and Drug Administration (FDA) and European Medicines Agency (EMA) guidelines (19, 20) and the European Bioanalysis Forum (EBF) recommendations (21, 22) for DBS assays. Furthermore, this study evaluates and validates the clinical applicability of this method by comparing paired DBS and plasma samples from 16 Ethiopian HIV-coinfected VL patients who received miltefosine treatment.

## MATERIALS AND METHODS

### Chemicals and reagents.

**FIG 1:**
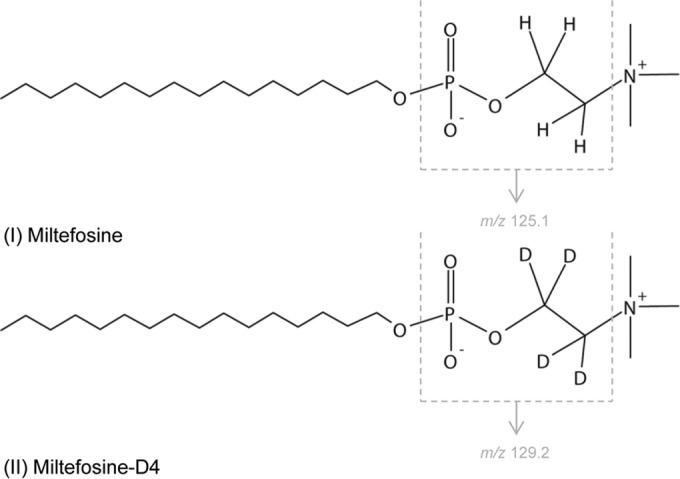
Structural formulas of miltefosine and the internal standard miltefosine-D4, indicating the *m/z* fragments.

Miltefosine was purchased from Sigma-Aldrich (Zwijndrecht, the Netherlands). Deuterated miltefosine (miltefosine-D4) (Fig. 1) was purchased from Alsachim (Illkirch Graffenstaden, France). Methanol and water were obtained from Biosolve Ltd. (Valkenswaard, the Netherlands). Ammonia (25%) was purchased from Merck (Amsterdam, the Netherlands).

### Materials.

For the collection of DBS samples, pure cellulose-based cards (Whatman 903 protein saver cards) were used. These cards, together with foil bags and desiccant packages for storage of DBS samples, were purchased from GE Healthcare Europe GmbH (Diegem, Belgium). A Harris 3.0-mm micropunch was used to punch the DBS samples. Whole blood (WB) was collected in K_2_EDTA BD Vacutainers from healthy volunteers and stored at 2 to 8°C for a maximum of 2 days. WB was adjusted to a Ht level of 30% ± 1% (Ht30 WB), to mimic the Ht levels of VL patients, by dilution with plasma. Ht levels were determined with the Cell Dyn Hematology analyzer (Abbott Diagnostics, Lake Forest, IL, USA).

### Preparation of calibration standards and QC samples.

Stock solutions of 1 mg/ml miltefosine were prepared from independent weighings in methanol-water (1:1 [vol/vol]). Separate stocks were diluted to working solutions with methanol-water (1:1 [vol/vol]) for the preparation of calibration standards and quality control (QC) samples. A stock solution of 1 mg/ml deuterated miltefosine (miltefosine-D4) was prepared and diluted to an internal standard (IS) working solution of 4,000 ng/ml miltefosine-D4 in methanol-water (1:1 [vol/vol]). This working solution was further diluted with methanol to an extraction solution of 20 ng/ml miltefosine-D4 in 100% methanol. The stock and working solutions were stored at nominally −20°C.

Calibration standards were diluted 1:20 (vol/vol) in Ht30 WB to final concentrations of 10, 20, 100, 500, 1,000, 1,400, 1,800, and 2,000 ng/ml. QC samples were diluted 1:20 (vol/vol) in Ht30 WB to final concentrations of nominally 10, 24, 300, and 1,600 ng/ml (lower limit of quantification [LLOQ], low-level QC [QCL], mid-level QC [QCM], and high-level QC [QCH], respectively). Additionally, a sample above the upper limit of quantification (>ULOQ), i.e., 40,000 ng/ml, was prepared and used to determine dilution integrity.

A volume of 20 μl of spiked whole blood was spotted on Whatman 903 cards and air dried for at least 3 h at room temperature. When samples that had been dried for 3 h were compared to samples that had been dried overnight (15 to 20 h), no effect was found for the additional drying time (bias within ±6.1%).

### Sample pretreatment.

After drying, a 3.0-mm punch was taken from the center of the DBS and transferred to a 1.5-ml Eppendorf tube. To prevent spot-to-spot puncher carryover, an unspotted filter punch was taken after each sample punch. A total of 150 μl of extraction solution (20 ng/ml miltefosine-D4 in methanol) was added to each sample with the exception of double blanks, to which 150 μl of methanol was added. The tubes were mixed for 10 s, sonicated for 30 min, and mixed for another 30 s. Subsequently, the final extract was transferred to an autosampler vial, and 10 μl was injected onto the high-performance liquid chromatography (HPLC) column. No additional recovery of miltefosine from the blood spots was found when longer sonication times were used.

### Liquid chromatography-tandem mass spectrometry.

Chromatographic separation was performed as described for the previously validated miltefosine plasma method (3), using a Gemini C_18_ precolumn (4.0 mm by 2.0-mm inside diameter [i.d.]; Phenomenex, Torrance, CA, USA) and Gemini C_18_ analytical column (150 mm by 2.0-mm i.d.; particle size, 5 μm; Phenomenex), with isocratic elution with 10 mM ammonia in 95% methanol (vol/vol) at 0.3 ml/min. The HPLC system (Agilent 1100 series; Agilent, Palo Alto, CA, USA) consisted of a binary pump, in-line degasser, autosampler (at 4°C), and column oven (at 25°C). The miltefosine concentrations were analyzed on an API-3000 triple-quadrupole mass spectrometer (MS) equipped with a turbo-ion-spray source (Sciex, Framingham, MA, USA), operating in positive ion mode. Table 1 summarizes the MS operating parameters.

**TABLE 1:** MS operating parameters for determination of miltefosine in dried blood spots

Parameter	Miltefosine	Miltefosine-D4
Run duration (min)	5.0	5.0
Ion-spray voltage (kV)	+4.5	+4.5
Turbo gas temperature (°C)	400	400
Turbo gas flow (liters/min)	7	7
Nebulizer gas pressure (arbitrary units)	11	11
Curtain gas pressure (arbitrary units)	9	9
Collision gas pressure (arbitrary units)	6	6
Parent mass (*m/z*)	408.5	412.6
Product mass (*m/z*)	125.1	129.2
Dwell time (ms)	400	400
Collision energy (V)	43	43
Collision exit potential (V)	22	22
Declustering potential (V)	71	71
Focusing potential (V)	290	290
Entrance potential (V)	12	12
Typical retention time (min)	2.6	2.6

### Validation of assay for quantification of miltefosine in DBS samples.

The validation of the assay was performed according to the most current EMA and FDA guidelines for the validation of bioanalytical assays (19, 20), with respect to the following aspects: calibration model, accuracy and precision, LLOQ, selectivity (endogenous interferences and cross-analyte interferences), carryover (instrumentation and spot-to-spot carryover), dilution integrity, matrix effects, and recovery. Additional experiments were performed for the application of dried blood spots as a matrix according to EBF recommendations (21, 22); blood spot volume, blood spot homogeneity, and different WB Ht values were tested for their effects on accuracy and precision at two concentrations (QCL and QCH). Stability for up to 162 days was tested at four nominal temperatures, i.e., −70°C, −20°C, room temperature (20 to 25°C), and 37°C.

### Clinical application.

As part of a larger randomized clinical trial (ClinicalTrials registration no. NCT02011958) investigating the treatment of Ethiopian HIV-coinfected VL patients with high-dose liposomal amphotericin B alone (total dose of 40 mg/kg, given over 24 days) or liposomal amphotericin B (total dose of 30 mg/kg, given over 11 days) in combination with a 28-day miltefosine regimen (2.5 mg/kg daily), paired plasma and DBS samples were collected from 16 patients. Ethical approval was obtained from the Ethiopian National Research Ethics Review Committee, the institutional review board of the University of Gondar in Ethiopia, and ethics committees from Médecins Sans Frontières, the London School of Hygiene & Tropical Medicine, and the Institute of Tropical Medicine (Antwerp, Belgium). Regulatory approval was obtained from the Food, Medicine, and Health Administration and Control Authority in Ethiopia. All patients provided written informed consent before entering the study. DBS and plasma samples were collected simultaneously on day 29 of miltefosine treatment, 1 day after the last miltefosine dose, when patients are considered to have reached steady-state/maximal levels.

Plasma samples were collected using K_2_EDTA BD Vacutainers; after centrifugation, plasma was isolated and was maintained at −20°C until analysis. DBS samples were collected from a finger-prick using a lancet (GST Corp., New Delhi, India). A drop of blood was applied to a Whatman 903 protein saver card without touching the filter paper with the finger tip. DBS samples were allowed to air dry for at least 3 h before being stored in an airtight and watertight zipper-lock bag containing at least three desiccant packages. DBS samples were stored and transported by courier at room temperature. Ht levels of the patients were determined with a Beckman Coulter AcT Diff hematology analyzer (Beckman Coulter, Fullerton, CA, USA).

Observed DBS and plasma concentrations were compared using weighted Deming regression, and a Bland-Altman difference plot was used to depict the agreement between the two methods. All statistical analyses were performed with R (version 3.1.2). The acceptance criteria for the agreement between the observed and derived plasma concentrations were based on the guideline for incurred sample reanalysis of the EMA, i.e., the difference between the observed and derived miltefosine plasma concentrations should be within ±20% for at least 67% of the samples (19).

## RESULTS

### Calibration model.

Calibration standards at eight concentrations in the range of 10 to 2,000 ng/ml were prepared and analyzed in duplicate on 3 separate days at the beginning and end of the analytical run. To obtain the lowest total bias across the range, the linear regression of the analyte/internal standard peak area ratio (AR) versus the concentration of miltefosine (*x*) was weighted, 1/*x*^2^. The calibration curve was accepted if 75% of the nonzero calibration standards were within ±15% of their nominal concentrations (±20% for the LLOQ). At least one calibration standard at the LLOQ and upper limit of quantification (ULOQ) should be accepted. All three calibration curves met these criteria and had correlation coefficients (*R*^2^) of ≥0.9964.

### Accuracy and precision.

The accuracy and precision of the method were determined by analyzing the LLOQ, QCL, QCM, and QCH five times in three separate analytical runs. Intra-assay and interassay bias values were within ±15% of the nominal concentrations for all QC samples. As presented in Table 2, intra-assay and interassay precision values (expressed as coefficient of variation [CV] values) were ≤7.0% for QCL, QCM, and QCH and ≤19.1% for the LLOQ. Therefore, both the accuracy and precision of the method were found to be acceptable.

**TABLE 2:** Intra-assay and interassay accuracy (bias) and precision (CV) determined by analyzing quality control samples at four concentrations, i.e., LLOQ (10.1 ng/ml), QCL (24.2 ng/ml), QCM (302 ng/ml), and QCH (1,610 ng/ml)

Parameter	Nominal concn (ng/ml)	Bias (%)	CV (%)	No. of replicates
LLOQ				
Run 1	10.1	−9.0	9.1	5
Run 2	10.1	8.1	7.1	5
Run 3	10.1	4.8	19.1	5
Interassay	10.1	1.3	14.3	15
QCL				
Run 1	24.2	1.2	5.8	5
Run 2	24.2	6.3	4.6	5
Run 3	24.2	1.1	6.5	5
Interassay	24.2	2.8	5.8	15
QCM				
Run 1	302	2.0	1.3	5
Run 2	302	−2.5	3.6	5
Run 3	302	11.2	6.2	5
Interassay	302	3.6	7.0	15
QCH				
Run 1	1,610	−0.5	5.4	5
Run 2	1,610	0.9	5.2	5
Run 3	1,610	5.5	3.5	5
Interassay	1,610	1.9	5.1	15

### Lower limit of quantification.

**FIG 2:**
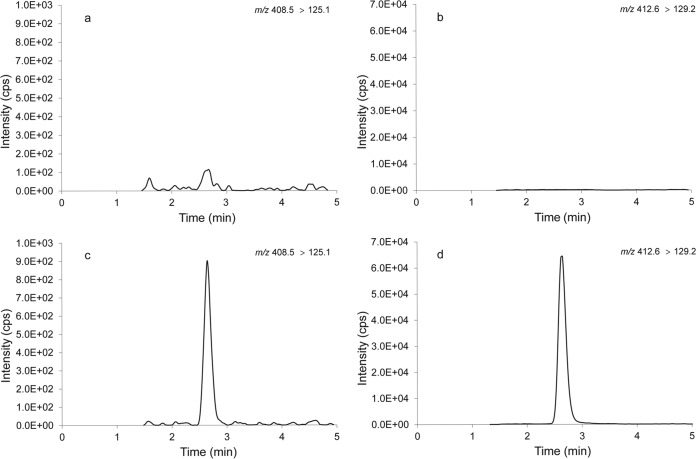
Representative LC-MS/MS ion chromatograms of miltefosine (a) and the internal standard miltefosine-D4 (b) quantified in a double-blank DBS sample and of miltefosine (c) and miltefosine-D4 (d) in an LLOQ sample (10.1 ng/ml).

The first blank and the five LLOQ quality control samples were used to determine the signal-to-noise ratio in three analytical runs. The signal-to-noise ratio of miltefosine at the LLOQ level was above 5 for all three runs (i.e., 9.6, 5.3, and 5.8). Figure 2 shows representative LC-MS/MS ion chromatograms for miltefosine and the internal standard in a double-blank sample and an LLOQ sample.

### Specificity and selectivity.

Six different batches of human WB were collected from six healthy donors and adjusted to Ht30 WB, and both a double-blank sample and an LLOQ sample were prepared from each batch. The samples were processed and analyzed as described above. The six LLOQ samples were all within ±20% of their nominal values. For the double-blank samples, five of the six batches showed no interference at the retention time of miltefosine over 20% of the peak area of the LLOQ sample and none showed a peak for miltefosine-D4 higher than 5% of the internal standard peak area. Therefore, the selectivity was considered to be sufficient.

To test the cross-analyte interference, an ULOQ sample was prepared as described above but subsequently processed with the addition of methanol as the extraction solvent (without the internal standard). Additionally, the internal standard was spiked separately in a double-blank sample at the nominal concentration. No internal standard interferences were observed for the analyte signal, and no interference from the analyte was measured for the specific mass transition of the internal standard.

### Dilution integrity.

The mean miltefosine concentration at the end of a 28-day treatment (150 mg/day) was found to be ∼30,000 ng/ml in Dutch CL patients (17). Therefore, an >ULOQ sample of 40,000 ng/ml was used in the dilution integrity experiment. The >ULOQ sample was prepared as described previously, and the final extract was subsequently diluted 100-fold with the final extract of a processed blank DBS (extracted with extraction solvent containing the internal standard). The dilution steps were as follows: first, 10 μl of >ULOQ final extract was diluted with 90 μl of blank final extract; subsequently, 10 μl of this dilution was further diluted with another 90 μl of blank final extract. The deviations of the diluted >ULOQ samples were within ±3.3% of the nominal concentration, and the precision was ≤2.0%; therefore, it was concluded that samples exceeding the ULOQ (up to 40,000 ng/ml) could be diluted as described, applying a dilution factor of 100.

### Carryover.

Two types of carryover are important to investigate in the validation of dried blood spot methods, namely, instrument carryover and spot-to-spot carryover caused by the punching device. These two sources of carryover were tested. Spot-to-spot carryover samples were prepared by punching spots in the following sequence: an ULOQ sample, unspotted filter paper (to eliminate most of the carryover), a blank spot, unspotted filter paper, and a blank spot. The two blank spots were processed as described previously and injected after the ULOQ sample. The combined instrument and spot-to-spot carryover of the two samples was compared to the mean value of five LLOQ sample measurements and was found to be below 19.3% of the LLOQ.

However, in clinical practice, miltefosine concentrations are often expected to exceed the calibration range of 10 to 2,000 ng/ml. Samples with expected concentrations around the ULOQ or >ULOQ values should preferably be analyzed in one batch. After punching of a 40,000-ng/ml >ULOQ sample, carryover is acceptable (<20% of LLOQ) at the fourth blank spot punched subsequently.

### Matrix factor and recovery.

The matrix factor (MF) and recovery were tested in six different batches of Ht30 WB, spiked at QCL and QCH singularly. Ten-microliter spots were prepared from these solutions, so-called “processed DBS samples.” For the analysis, the entire spot was cut out and processed as described previously, with 150 μl of extraction solvent. Additionally, “matrix-absent” and “matrix-present” samples were prepared, for which two neat solutions, namely, MF-low (MF-L) (24.4 ng/ml miltefosine) and MF-high (MF-H) (1,630 ng/ml miltefosine), were first prepared in extraction solvent (20 ng/ml miltefosine-D4 in methanol). The matrix-absent samples were prepared by diluting 10 μl of these neat solutions with 140 μl of extraction solvent. The matrix-present samples were prepared by cutting out the entire 10-μl blank spots of the six different Ht30 WB batches, after which 10 μl of MF-L or MF-H solution and 140 μl of extraction solvent were added.

The MF was calculated for each batch by calculating the ratio of the miltefosine peak area in the matrix-present sample to that in the matrix-absent sample. The MF at both tested concentrations was ∼0.3 as a result of matrix effects (ion suppression). The IS-normalized MF was ∼1.0, which indicated that the stable isotope-labeled internal standard was effectively compensating for any matrix effects. At both tested QC levels, the CVs of the absolute and IS-normalized MF values calculated from the six different Ht30 WB batches were below 11.5%.

Given that the internal standard is added as extraction solution, it is not part of the sample pretreatment; therefore, the IS-normalized values were used to determine recovery. The sample pretreatment recovery was calculated by comparing the area ratio (AR) of the processed DBS samples with the AR of the matrix-present samples. IS-normalized sample pretreatment recovery was ∼100% (97.2% for QCL samples and 103% for QCH samples). At both tested QC levels, the CV of the IS-normalized recovery from the 6 batches was below 6.7%. Both the matrix effect and recovery experiments were considered acceptable, because the CVs calculated for the six different Ht30 WB batches were consistent and below 15%.

### Stability.

DBS QC samples were prepared at two concentrations (QCL and QCH), as described previously, and were air dried at room temperature overnight. The following day, the samples were stored in sealed aluminum bags with three desiccant packages at four temperatures, i.e., −70°C, −20°C, room temperature (20 to 25°C), and 37°C. Stability was tested on days 34, 58, 107, and 162; the measured concentrations were within ±12.5% of the nominal concentrations and the precision was ≤10.7%. The stability of miltefosine in DBS samples was proven to be at least 5 months (162 days) at temperatures ranging from −70°C to 37°C, with storage in sealed aluminum bags with three desiccant packages.

### Blood spot homogeneity.

Blood spot homogeneity was investigated with 20-μl Ht30 WB DBS samples at QCL and QCH levels, in triplicate; 3.0-mm punches were taken at the perimeter instead of the center of the spots. The bias was 21.8% for the QCL level and 18.0% for the QCH level (CV, ≤7.4%), which points out the importance of punching the center of the spot.

### Effect of blood spot volume.

For all of the validation procedures described here, a standard fixed spot volume of 20 μl was used. QCL and QCH samples were spotted in blood spot volumes reflecting the procedure in clinical practice, i.e., 10, 15, 25, and 30 μl. Samples were analyzed in triplicate. Accuracy and precision were all within ±13.4%, indicating that variability in blood spot volumes between 10 and 30 μl had no effect on the accuracy and precision of the method (data not shown).

### Effect of hematocrit levels.

Human WB was adjusted to a range of Ht values that were expected in clinical practice with HIV-coinfected VL patients, i.e., 20, 23, 31, and 35%. For each Ht level, QCL and QCH samples were spiked and analyzed in triplicate. The accuracy and precision of DBS samples within this Ht range were all within ±14.1% and ≤7.2%, respectively, and therefore were considered acceptable (within ±15%) (data not shown). However, a linear effect of Ht values on the miltefosine quantification was visible in these experiments; therefore, a wider range of Ht values was prepared, to investigate the relationship between Ht levels and the bias in miltefosine quantification. Human WB was adjusted to five different Ht levels (10, 21, 30, 40, and 51%), spiked at two concentrations (QCL and QCH), and spotted at a volume of 20 μl. Samples were analyzed in triplicate.

**FIG 3:**
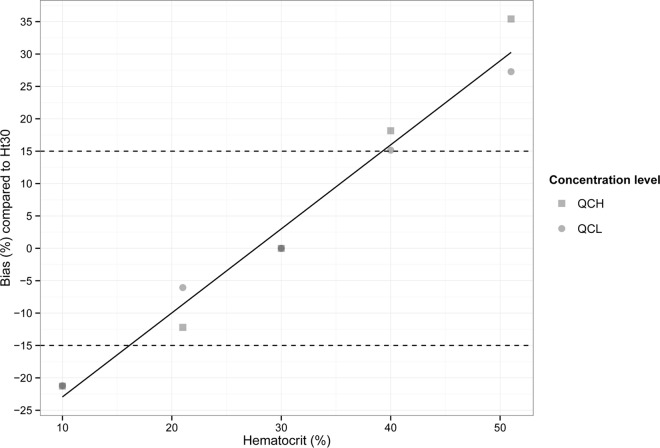
Effects of hematocrit levels on the accuracy of miltefosine quantification at two concentrations (i.e., QCL [24 ng/ml] and QCH [1,600 ng/ml]), depicted as bias in the area ratio in comparison with Ht30 WB (used for calibration standards). The linear regression line is described as bias_Ht_ = [(0.013 × Ht) − 0.359] × 100%. Dashed lines, 15% bias.

Figure 3 depicts the bias caused by Ht levels in the area ratio of quality control samples prepared in WB with different Ht levels, relative to standard quality control samples prepared in WB Ht30. The linear trend in the bias of the miltefosine concentrations with increasing Ht levels relative to Ht30 could be described by equation 1 (*R*^2^ = 0.9761).

(1) $${\text{Bias}}_{\text{Ht}}=[(0.013\times \text{Ht)}-0.359]\times 100\%$$

The same Ht range was spotted at 10, 30, 40, and 50 μl, and the linear regression had approximately the same slope regardless of the blood spot volume (data not shown).

### Clinical evaluation of DBS versus plasma concentrations in patient samples.

**FIG 4:**
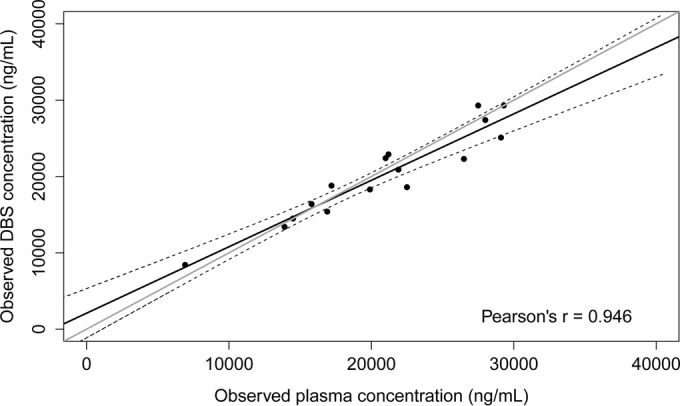
Observed miltefosine dried blood spot concentrations plotted against the corresponding observed plasma concentrations in paired patient samples (*n* = 16). Solid black line, weighted Deming fit (2,091 + 0.87*x*; Pearson's *r* = 0.946); dashed black lines, 95% confidence interval of the fit; solid gray line, line of true identity.

A total of 16 paired DBS and plasma samples were available from miltefosine-treated Ethiopian HIV-coinfected VL patients. Samples originated from the last treatment day, at which miltefosine plasma concentrations exceed the ULOQ. Miltefosine concentrations ranged from 8,420 to 29,300 ng/ml and from 6,920 to 29,300 ng/ml for DBS and plasma samples, respectively. The median of the observed miltefosine DBS/plasma concentration ratio was 0.99 (range, 0.83 to 1.22). The correlation between paired individual observed miltefosine plasma and DBS concentrations, using a weighted Deming regression, is depicted in Fig. 4. The slope of the weighted regression line was 0.87 (95% confidence interval [CI], 0.70 to 1.04), with an intercept of 2,091 (95% CI, −1,132 to 5,313) (Pearson's *r* = 0.946). The line of true identity, with a regression slope of 1, lies within the 95% CI of the Deming regression line (Fig. 4). This indicates an approximately equal distribution of miltefosine in blood plasma and erythrocytes.

Miltefosine (MIL) plasma concentrations can thus be derived from the observed DBS concentrations by using the derived Deming regression equation, as follows:

(2) $${\left[\text{MIL}\right]}_{\text{plasma},\text{derived}}=\frac{\left({\left[\text{MIL}\right]}_{\text{DBS}}-2,091\right)}{0.87}$$

**FIG 5:**
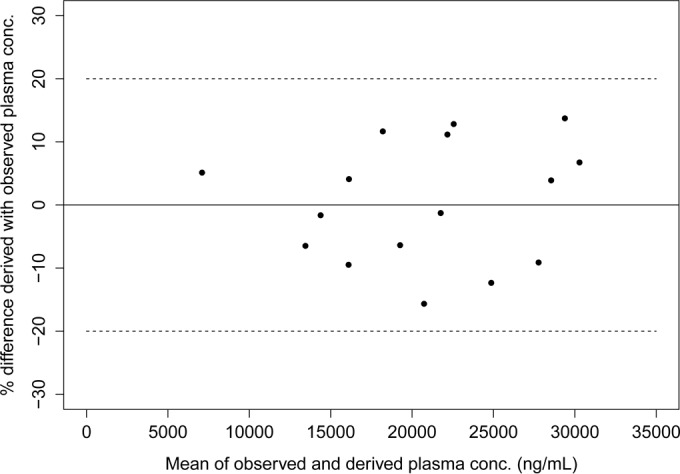
Bland-Altman difference plot depicting the differences between the plasma concentrations derived using the Deming regression equation, based on the observed DBS concentrations, and the observed plasma concentrations. Dashed lines, 20% bias, compared to the observed plasma concentrations.

All derived miltefosine plasma concentrations calculated from the observed DBS concentrations by using equation 2 were within ±20% of the observed plasma concentrations, as shown in the Bland-Altman plot (Fig. 5).

Large between-patient variability in baseline Ht levels is expected for VL patients, and Ht levels typically increase over time during the treatment period as patients recover from their infections. Given the effect of Ht levels on the miltefosine quantification with DBS samples established in the bioanalytical validation, the appropriateness of Ht correction of the clinical DBS concentrations was assessed using the patients' paired DBS and plasma samples. Individual patient Ht levels were available for all paired samples, ranging between 23.4% and 44.0%, with a median of 30.5%. We tested Ht correction of the observed DBS concentrations for these clinical samples by using equation 1, describing the effect of Ht levels on miltefosine quantification in the bioanalytical validation, which resulted in equation 3.

(3) $${\left[\text{MIL}\right]}_{\text{DBS},\text{corrected}}=\frac{{\left[\text{MIL}\right]}_{\text{DBS},\text{observed}}}{0.641+(0.013\times \text{Ht})}$$

**FIG 6:**
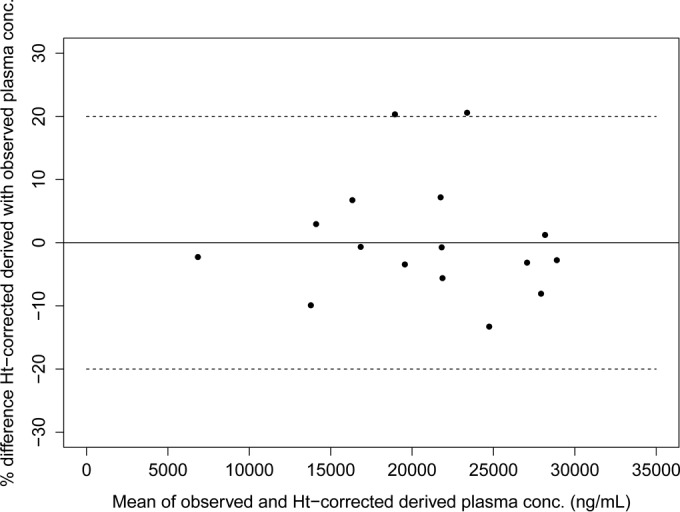
Bland-Altman difference plot depicting the differences between the Ht-corrected plasma concentrations derived using the Deming regression equation, based on the Ht-corrected DBS concentrations, and the observed plasma concentrations. Dashed lines, 20% bias, compared to the observed plasma concentrations.

**FIG 7:**
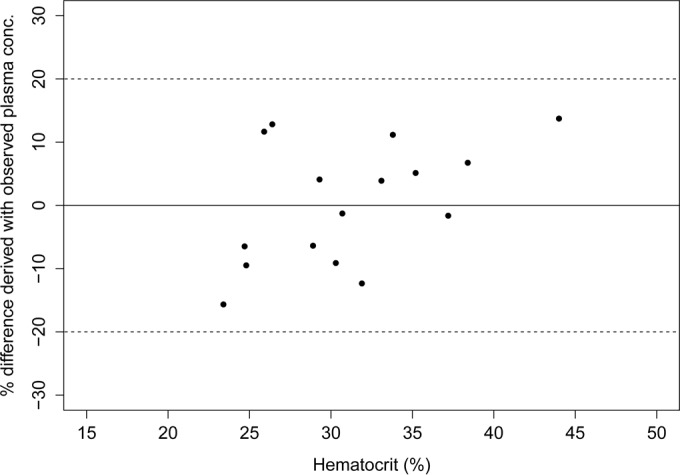
Differences between the plasma concentrations derived using the Deming regression equation, based on the observed DBS concentrations (without Ht correction), and the observed plasma concentrations versus hematocrit levels. Dashed lines, 20% bias, compared to the observed plasma concentrations.

The correlation between the Ht-corrected DBS concentrations and the corresponding observed plasma concentrations using a weighted linear Deming regression resulted in a slope of 0.83 (95% CI, 0.73 to 0.94), with an intercept of 2,051 (95% CI, 238 to 3,863) (Pearson's *r* = 0.951) (graph not shown). The 95% CIs of both the slopes and intercepts of the Ht-corrected and non-Ht-corrected regression lines were overlapping, indicating that Ht correction does not provide a significantly better fit. While all derived plasma concentrations were within 20% of the observed plasma concentrations without Ht correction, 2 of the 16 paired samples were outside the ±20% bias, relative to the observed plasma concentrations, when the DBS concentrations were first corrected for Ht bias (Fig. 6). Furthermore, no obvious or systematic trend in the bias of the derived plasma concentrations (no Ht correction) versus Ht levels was visible (Fig. 7). Based on the clinical validation, correction of miltefosine DBS concentrations for Ht levels appeared not to be appropriate.

## DISCUSSION

The assay described here is the first assay to measure miltefosine concentrations in patients using less-invasive DBS sample collection, to facilitate future clinical trials investigating new antileishmanial treatment regimens including the drug miltefosine. The assay was successfully validated according to FDA/EMA guidelines and EBF recommendations. With this method, miltefosine can be accurately and precisely quantified with an LLOQ of 10 ng/ml, and concentrations as high as 40,000 ng/ml can be analyzed by 100-fold dilution. Paired miltefosine DBS and plasma samples were collected from 16 HIV-coinfected VL patients in Ethiopia. This clinical evaluation demonstrated good correlation between observed plasma and DBS concentrations. Miltefosine plasma concentrations derived from the observed DBS concentrations using a weighted Deming regression were within 20% of the observed plasma concentrations over a wide range of concentrations. We showed here that the observed miltefosine DBS concentrations were approximately equal to the paired observed plasma concentrations. This indicates an equal distribution of miltefosine between erythrocytes and plasma in blood of miltefosine-treated VL patients, an observation that has not been shown previously, to the best of our knowledge. DBS samples were found to be stable for at least 162 days at up to 37°C, using a simple storage procedure with desiccant packages, which enables storage of the miltefosine PK DBS samples at room temperature in tropical regions.

### Influence of Ht levels on miltefosine DBS measurements.

The patients included in our study showed variable Ht levels, as described previously (15), with a median of 30.5%, which is around the standardized Ht level of 30% used for the preparation of calibration standards and QC samples in this assay. Despite a linear correlation between Ht levels and the miltefosine DBS quantification bias observed during the laboratory bioanalytical validation, no such trend in bias due to Ht levels was found in the clinical application, with individual patients' Ht levels ranging from 23.4% to 44.0%. Ht correction did not significantly improve the calculation of the derived miltefosine plasma concentrations from the observed DBS concentrations in patient samples. Additionally, samples from 4 of 16 patients exceeded the validated Ht range (i.e., 35.2%, 37.2%, 38.4%, and 44.0%), but for those samples also the observed plasma concentrations were accurately described by the observed DBS concentrations, without the need for Ht correction.

These findings showed that the observed Ht effect on miltefosine quantification in the bioanalytical validation could not be confirmed in the clinical validation. Several factors can be hypothesized to have effects on miltefosine quantification in clinical practice, which together could potentially counteract the observed effect of Ht levels on miltefosine determinations. The most general explanation for the Ht effect on analyte quantification is that Ht levels affect the distribution of the applied blood over the filter paper (12). Blood with high Ht levels spreads less, and therefore the fixed-diameter subpunches contain larger volumes of blood than do samples of blood with lower Ht levels. It could be argued that, when the bioanalytical validation samples are spotted with a pipette, more pressure is applied than during finger-prick spotting, in which the drop merely falls onto the paper. This difference in blood flow upon application of the blood spot to the filter paper might theoretically reduce the total blood volume contained in the 3.0-mm punch from the dried blood spot.

It could also be hypothesized that the blood spot volume is larger for patients with lower Ht levels, due to lower viscosity of the blood leading to higher blood flow. However, when the blood spot diameters as indications of blood spot volumes (23) were compared for the blood spots in this clinical validation, no such trend between Ht levels and blood spot diameters was found for the patient samples (*R*^2^ = 0.002) (data not shown). Therefore, this is not likely to explain the absence of Ht-related bias in the miltefosine quantification of clinical samples.

Additionally, the DBS samples used in the bioanalytical validation differed from the clinical DBS samples in terms of matrix. While the clinical samples were derived from capillary blood obtained by finger puncture, venous blood obtained by venipuncture was used for bioanalytical validation purposes, for practical reasons. It was reported previously that the analyte concentrations in these two matrices could differ, which was mostly explained by the slower distribution equilibrium toward the capillaries (24). However, miltefosine accumulates during treatment and reaches steady-state levels during the last week of treatment for most patients. Because the clinical DBS samples were collected 1 day after the last dose of miltefosine, we did not expect the miltefosine concentration to differ between these two matrices.

Finally, during the bioanalytical validation, the effect of Ht was tested while other blood constituents, such as plasma proteins and other blood cells, were kept constant. In clinical samples, however, these blood constituents may be variable and potentially correlated with Ht levels, affecting the miltefosine quantification. For instance, serum albumin levels are significantly lower during active VL infections than those in healthy control subjects (25), as are Ht levels, and both anemia and low albumin levels were found to be risk factors for poor clinical outcomes in VL (26). Therefore, low Ht levels and low albumin levels are expected to be correlated. Miltefosine is highly protein bound (96 to 98%), and the majority of the protein-bound fraction (97%) is bound to albumin (27). This could imply that reduced serum albumin levels theoretically would lead to an increase in the unbound miltefosine fraction in plasma and correspondingly to increased distribution of miltefosine toward the erythrocytes (5). The effects of blood protein changes, concurrent with low Ht levels, on the quantification of miltefosine cannot be accounted for in the bioanalytical validation.

In conclusion, various clinical factors potentially affect miltefosine quantification, cancelling out the systematic bias caused by Ht levels and making individual Ht correction redundant in clinical practice. The absence of bias due to Ht levels in the clinical samples makes the application of DBS sample collection easier in the field, without the explicit need for concurrent Ht measurements, and thus allows for DBS sample collection without expensive laboratory equipment.

### Applicability of miltefosine DBS sampling method.

For the clinical validation, we had only a limited number of paired samples available. While there is no strict consensus regarding the number of paired samples required for method comparisons, the evaluation of 40 samples has been proposed (28). However, the collection of additional paired samples from the highly anemic HIV-coinfected patients in this study was unfortunately not feasible, due to practical limitations and ethical constraints. Paired patient samples were available over a wide but relatively high (>ULOQ) range of miltefosine plasma concentrations, between 6,920 and 29,300 ng/ml. However, as no trend could be observed concerning the effect of Ht levels on miltefosine quantification from DBS samples in clinical practice over this wide concentration range, we do not expect that Ht correction will be needed for lower concentration ranges.

We have demonstrated that DBS sample collection is a valid alternative to plasma sampling for the quantification of miltefosine, which has many practical advantages. DBS sampling is minimally invasive and requires only a minute volume of blood. This is particularly beneficial for application of the method in a pediatric population (a large proportion of VL patients are <12 years of age), as well as, for example, highly anemic HIV-coinfected VL patients. Additionally, DBS collection constitutes a low biohazard, reducing the risk of needle stick incidents when sampling HIV-coinfected VL patients. Finally, expensive and logistically challenging cold-chain storage and transport are not required for the DBS samples, simplifying the conducting of PK studies in remote areas where leishmaniasis is endemic and only limited clinical and laboratory infrastructure is available.
